# Recess in the 21st Century Post‐COVID World

**DOI:** 10.1111/josh.13235

**Published:** 2022-08-17

**Authors:** Global Recess Alliance, Catherine L. Ramstetter, Ed Baines, Charlene Woodham Brickman, Brendon Hyndman, Olga Jarrett, Rebecca A. London, William Massey, Lauren McNamara, Robert Murray, Debbie Rhea

**Affiliations:** ^1^ Successful Healthy Children, 629 Liddle Lane, Wyoming, OH 45215; ^2^ University College London, Institute of Education, 20 Bedford Way, London WC1H 0AL, UK; ^3^ Recess and Play, 145 Watson Drive, Athens, GA 30605; ^4^ Charles Sturt University Albury/Wodonga, Building 763, Room 133 North Wagga NSW 2650 Australia; ^5^ Georgia State University, Atlanta, 1070 Ashbury Drive, Decatur, GA 30030; ^6^ University of California, Santa Cruz, 1156 High Street, Santa Cruz, CA 95064; ^7^ Oregon State University, Women's Building 203C, 160 SW 26th Street, Corvallis, OR 97331; ^8^ Recess Project Canada 415 Yonge Street, 7th Floor, Suite 701, Toronto Ontario M5B 2E7 Canada; ^9^ American Academy of Pediatrics, 1629 Berkshire Road, Columbus, OH 43221; ^10^ Texas Christian University Annie Richardson Bass Building 2800 West Bowie Street, Fort Worth, TX 76109

The abrupt onset of the COVID‐19 pandemic forced the world into frenzied action, creating a series of ongoing stressors: school/work closings, remote learning, canceled events, family strife, fear, and a significant loss of social interactions. It is now unsurprising to learn that children's mental health has suffered. As social connection is tightly entwined with children's mental health, supporting school‐based spaces for quality social interactions is an important post‐pandemic recovery strategy. The unstructured school recess space is ideal for supporting recovery. A large and growing body of evidence supports the important role of recess, yet evidence also suggests that recess is not always implemented in ways that fulfill its promise.

We use “recess” as an inclusive term for meaningful, self‐directed, unstructured play at school for all children, through adolescence, ideally occurring outdoors. We note that what is referred to as recess by the American Academy of Pediatrics is referred to as “breaktime” in other countries. Despite advances in comprehensive school health, recess has received comparatively little attention with respect to translation of research findings, innovation, and change efforts. At a time of increased concern about the well‐being of children and adolescents, addressing this setting is an especially relevant area of inquiry for school health.

Recess offers the potential to positively shape learning, social connection, emotional well‐being, and physical health. When daily recess is available and with attention to creating safe and healthy play opportunities, research shows improvements in student attention, emotional regulation, classroom behavior, and overall school climate. Furthermore, less chaos and bullying at recess occur when safe and healthy play opportunities exist, which reduces the time teachers need to ready their students for learning when they return to class. Yet, recess is also a space that can be a challenge for schools, as a time when children may experience or witness negative social interactions such as bullying, isolation, or exclusion.

For recess to deliver its full potential, to be an inclusive, equitable space that alleviates stress, and promotes holistic child development, we must take action. The purpose of our commentary is to elevate school recess in the global conversation of schooling, specifically to highlight recess for critical reflection, consolidating contemporary research and providing recommendations for an urgently needed way forward.

## RECESS FOR THE 21ST CENTURY: *PROMOTING AND PROTECTING CHILDREN'S RIGHT TO PLAY*

From a historical perspective, schools were designed to prepare students for an industrial‐era workforce; the architecture, desk arrangements, daily routines, and focus on traditional classroom instruction reflect this ideology. Little attention was given to other types of learning activities—such as recess—which were considered ancillary and deprioritized accordingly with respect to funding and accountability. While there has been a notable movement towards an overall focus on well‐being and equitable learning environments, the recess setting is often overlooked in school improvement efforts. As a result, schoolyards continue to receive minimal resources and consideration—particularly those in low‐resource neighborhoods—and this is reflected in the commonly‐seen asphalt‐covered, barren schoolyard that does little to invite meaningful play, recreation, and social engagement.

The global community has long recognized that breaks and unstructured play are fundamental to children's physical, social, mental, and emotional development as well as central to their enjoyment and happiness. Play was considered so necessary to healthy development that in 1987, the *United Nations Convention on the Rights of the Child* (UNCRC) deemed it one of 54 fundamental human rights: specifically Article 31, *The Right to Play, Rest, and Leisure*. Article 31 states: “every child has the right to rest and leisure, to engage in play and recreational activities appropriate to the age of the child, and to participate freely in cultural life and the arts.”^1^

The purpose of the 54 articles of the UNCRC is to ensure children's basic rights are protected and promoted regardless of race, religion, or abilities. In 2013, the UN Committee on the Rights of the Child reviewed the progress of article 31 and found that, more than 30 years later, little investment has been made to protect and promote this right, including by schools. The Committee found: “where investment is made, it is in the provision of structured and organized activities, but equally important is the need to create time and space for children to engage in spontaneous play, recreation and creativity, and to promote societal attitudes that support and encourage such activity.”^2^ Notably, the Committee highlighted equity concerns in children's rights, with “girls, poor children, children with disabilities, indigenous children, children belonging to minorities,”^2^ of particular concern.

Given that most children spend a considerable portion of their developmental years in the school community, recess provides a unique space in which children and adolescents can exercise these fundamental rights.

### Recess: The Science Behind Play and Learning

Pioneering research methods have led to breakthroughs in our understanding of the neurophysiology of learning. Of importance to educators, student learning can be strengthened by regular breaks during the school day; time to allow memory traces formed in the classroom to be stabilized. Without these breaks, learning can be eroded by the inability to retain information. Recess affords the time for such breaks that are critical for learning and provides opportunities for physical activity and play, which are vital for cognitive development. Furthermore, research in both exercise science and child development indicates physically active play and creative play enhance executive functioning skills, which are predictive of both academic readiness and academic achievement. Enhancing physical activity levels in children increases inhibition, cognitive flexibility, and working memory as measured by both psychophysiological and behavioral indices, thereby making a substantial positive impact on classroom learning. Play also decreases stress, which has positive implications on memory, learning, behavior, and mental health, thereby addressing the holistic needs of children in schools.

### Recess: A Needed Opportunity for Educator Training and Development

Training those who supervise recess is crucial for creating a safe, healthy, and equitable play space. Within teacher education programs, trainee teachers rarely receive adequate, formalized learning in the value of recess and unstructured play. Teacher certification programs often focus on direct instruction pedagogies and sacrifice time to promote developmentally appropriate practice in the areas of social and emotional development. This unbalanced approach places too much emphasis on skills‐based learning while ignoring the needs of the whole child.

Another concern is that many existing teachers consider recess supervision an unwanted burden. When recess is viewed as merely a time for students to expend energy, it devalues the important learning that the unstructured recess environment can offer. Educators require improved knowledge of the learning that occurs when students are playing during recess, and their role in supporting it.

In addition to teachers, recess is often supervised by a largely untrained body of paraprofessionals. For example, in the UK “mid‐day assistants” are typically responsible for recess, and training, if provided, is often informal and sometimes little more than general conversations as the need arises. This means that adults have no explicit training on their role in supporting play and a positive recess environment. As such, they are left to their own assumptions about how to supervise recess, and are unprepared to diagnose and moderate what might be problematic or dangerous behaviors. Schools should consider providing formal training for all who will supervise recess.

Effective teacher preparation programs must begin to place a stronger emphasis on interdisciplinary child development. In doing so, social‐emotional facets, such as play, risk, resiliency, and creativity can be brought into the classroom and encouraged during recess with the next wave of trained educators. Incorporating play for pre‐service teachers can assist educators in refreshing their connections to play thereby strengthening play opportunities for the students they will serve. Furthermore, there is a need for principals and others in the administration to gain a deeper understanding of how play and recess/breaks support the needs of the whole child, at every age and grade level.

### Recess: An Opportunity for Age‐Appropriate Leisure and Play for All Students

Globally, many students experience recess throughout their schooling; however, this is not always the case. For example, in the United States, recess is often discontinued after elementary education (typically year 5 or 6). However, even for pre‐adolescents and adolescents, recess offers important opportunities to socialize and engage in self‐chosen activities with friends and peers. These are important for the development of social skills on which future relationships are based.

Research demonstrates that during this transition from elementary to secondary, students often become less active at school. Developmentally, older students may prefer experiences that incorporate increased socialization. A challenge for secondary schools is that the way children desire to play shifts, requiring changes in the physical environment for recess. It is important to have well‐resourced spaces dedicated to specific activities with socializing opportunities (eg, games, art, dance). These may include multiple alternative supervised spaces in addition to the outdoor school yard, such as the library, art room, computer lab, and so on. To better accommodate recess opportunities for older students, we recommend schools consult with students on what their interests and needs are.

### Recess: An Opportunity to Recognize and Contest Inequity

Every child has the right to breaks in the school day, time in which to play, be active, and interact with their peers. However, research shows that there is disparity in recess quantity and quality by race/ethnicity, disability, and socio‐economic status. Children of color and those attending under‐resourced schools experience lower quality and fewer minutes of recess, a phenomenon documented in Australia, Canada, the UK, and the United States.

Other areas of concern are the limitations in play opportunities for children with disabilities. For example, many US school playgrounds are minimally compliant with current Americans for Disability Act regulations. Schools worldwide must consider play materials that provide appropriate adaptive physical and social play opportunities for children of all ages and abilities, which allow children with disabilities to interact with their classmates.

One challenge to recess equity is that it is common for students to miss part or all of recess due to poor behavior in class or on the playground, or to complete classwork or homework. Children who struggle behaviorally or academically at school are the ones most likely to have recess withheld thus missing the opportunities to learn from engagement with peers in games and play. Using recess deprivation as punishment is unlikely to lead to increased educational engagement and is counterproductive to the goals of whole child education. Positive, motivational approaches should become part of a formal school policy on recess.

### Recess Policy: An Opportunity to Elevate the Value of Play

Considering the directive of the UNCRC, there are several policy considerations to ensure students have access to daily recess. Policies can be made at the school or district level; however, state/provincial or national policy is the most effective way to reach the largest number of students, ensuring a safe and healthy recess experience. When crafting recess policy, we outline 5 policy parameters:

1. Secure time for daily recess. Key questions that arise are how much time and for which grade levels. We believe all children need one or more daily breaks. We recommend these breaks total no less than 40 minutes per day, broken into a minimum of 2 recess periods.
2. Disallow the withholding of recess for missed schoolwork or misbehavior. Although withholding recess has not been demonstrated in research as an evidence‐based practice, many teachers, administrators, lunch monitors, and recess supervisors continue to use withholding recess as both a threat and a punishment.
3. Provide training for teachers and paraprofessional staff that ensures safe, healthy, and inclusive recess. Training promotes buy‐in for recess.
4. Require data collection and reporting. Currently, there is no repository of information about whether schools offer recess, the time allotted for recess, or about recess withholding.
5. Include recommendations for a variety of equipment and loose parts (balls, hula hoops, jumpropes, painted lines on hard surfaces, safe surfaces, climbing equipment, etc.) considering the needs of children of all ages and abilities.

We also recommend accountability mechanisms to support compliance and effective policy implementation.

### Recess: Opportunity for the Future

Our vision is that researchers, educators, and policy makers respond to our call to action, with collaboration, expanding the current body of knowledge of the benefits of recess; examining barriers and best practices in delivering a safe, inclusive recess; and advocating for change based on these best practices—all essential to ensuring recess delivers its potential for all children to experience their right to play. It is long past time for recess to join education in the 21st century; more importantly, it is time for education to reclaim its purpose to teach skills, provide intellectual exploration and foster emotional development. Recess must be included in every educational decision, considering, and promoting what is in the best interest of the child: the right to rest, leisure and play.

### Conflict of Interest

The authors declare no conflicts of interest.
